# An unusual case of chronic meningitis

**DOI:** 10.1186/1471-2296-5-21

**Published:** 2004-10-06

**Authors:** Christopher Boos, Cyrus Daneshvar, Anna Hinton, Matthew Dawes

**Affiliations:** 1 Department of General Medicine, Portsmouth Hospitals NHS Trust, Milton Rd, Portsmouth, UK; 2 Department of Elderly Care Medicine, Portsmouth Hospitals NHS Trust, Milton Rd, Portsmouth, UK

## Abstract

**Background:**

Chronic meningitis is defined as symptoms and signs of meningeal inflammation and persisting cerebrospinal fluid abnormalities such as elevated protein level and pleocytosis for at least one month.

**Case presentation:**

A 62-year-old woman, of unremarkable past medical history, was admitted to hospital for investigation of a four-week history of vomiting, malaise an associated hyponatraemia. She had a low-grade pyrexia with normal inflammatory markers. A CT brain was unremarkable and a contrast MRI brain revealed sub-acute infarction of the right frontal cortex but with no evidence of meningeal enhancement. Due to increasing confusion and patient clinical deterioration a lumbar puncture was performed at 17 days post admission. This revealed gram-negative coccobacilli in the CSF, which was identified as *Neisseria meningitidis *group B. The patient made a dramatic recovery with high-dose intravenous ceftriaxone antibiotic therapy for meningococcal meningitis.

**Conclusions:**

1) Chronic bacterial meningitis may present highly atypically, particularly in the older adult. 2) There may be an absent or reduced febrile response, without a rise in inflammatory markers, despite a very unwell patient. 3) Early lumbar puncture is to be encouraged as it is essential to confirm the diagnosis.4) Despite a delayed diagnosis appropriate antibiotic therapy can still lead to a good outcome.

## Background

Bacterial Meningitis usually presents as an acute illness, predominantly affecting children and young adults. It typically presents with the classical clinical triad of fever, neck stiffness and an altered mental state. However, it may rarely present as a chronic illness, without the classic clinical features noted in acute meningitis.

## Case presentation

A 62-year-old retired woman was admitted to hospital via her GP for investigation of a four-week history of vomiting and malaise associated with hyponatraemia. She was initially diagnosed as suffering from viral gastroenteritis. However, the vomiting had persisted and had become associated with a mild frontal headache. She had an unremarkable past medical history and was not taking any regular medication. She had never smoked and there was no recent antecedent foreign travel.

On examination she appeared clinically dehydrated but otherwise looked well, and was alert and orientated. She was apyrexial and had no rash, photophobia, neck stiffness or stigmata of endocarditis. She had a sinus tachycardia of 104/minute, with normal heart sounds, and a blood pressure of 130/76 mmHg. Chest, abdominal and neurological examinations were unremarkable. She had a plasma sodium of 127 mmol/L (135–145 mmol/L), potassium of 3.4 mmol/L (3.5–5.0 mmol/l), urea 4.8 mmol/L (3.0–6.5 mmol/L) and creatinine of 68 mmol/L (60–125 mmol/L). There was no biochemical evidence of the syndrome of inappropriate antidiuretic hormone (SIADH) production (serum osmolality 261 mmols/kg; urine osmolality 71 mmols/Kg; urine sodium < 10 mmol/L). Serum complement and plasma immunoglobulin levels were unremarkable with no evidence of immunosuppression. In addition, she had a normal full autoimmune profile and thyroid function. Random cortisol level was mildly elevated at 799 nmol/L (normal 140 – 700 nmol/L) consistent with a stress response.

Her initial white cell count (WCC) was mildly elevated at 13.0 × 10^9^/L (normal 4–11 × 10^9^/L) with a neutrophilia of 10 × 10^9^/L (normal 2–7.5 × 10^9^/L). Her ECG and chest X-ray were normal. Her C-reactive protein (CRP) was slightly elevated at 10 mg/L (normal <5 mg/L) and erythrocyte sedimentation rate (ESR) was normal at 5 mm/hour. Her Chest X-ray and electrocardiogram were normal. Initial microbiological investigations (blood cultures, urine microscopy and culture) were normal.

Initial management consisted of slow intravenous rehydration with normal saline and antiemetic therapy, which led to a mild symptomatic improvement. Upper gastrointestinal endoscopy revealed mild oesophagitis.

During the ensuing two weeks her laboratory investigations remained stable (CRP normal; ESR normal; sodium 127–131 mmol/L; WCC 11–13 × 10^9^/L). However, on day 4 of admission she developed a low-grade pyrexia of 37.5°C, which persisted (<38°C). A CT scan of the head revealed periventricular patchy white matter changes but no features of raised intracranial pressure or space occupying lesion.

Unfortunately the patient had become slowly more lethargic, withdrawn, and depressed. By day 17 of admission, although alert, she was uncooperative with intermittent confusion. Her symptoms of intermittent nausea and vomiting with occasional frontal headache continued.

On day 18 she underwent a lumbar puncture (LP) as she still had a low-grade pyrexia (temperature 37.5°C) and neutrophilia of 9.3 × 10^9^/L). In addition, her nausea and vomiting had failed to fully settle with supportive treatment. The LP results were as follows: cerebrospinal fluid (CSF) appearance was pale yellow and clear; protein = 5.69 g/L (0.15–0.4 g/L); CSF glucose 1.7 mmol/L versus plasma glucose 5.7 mmol/L (ratio = 30%, normal > 50%); CSF WCC = 106/mL (normal <5 WCC/mL) – 99% lymphocytes. Gram's stain revealed gram-negative coccobacilli; acid-fast bacilli were not seen. She was commenced on intravenous ceftriaxone.

Contrast MRI brain revealed sub-acute infarction of the right frontal cortex but with no evidence of meningeal enhancement. EEG demonstrated slow wave activity, which was consistent with a meningo-encephalitis.

Within 48 hours of intravenous antibiotics she was more alert, orientated, and sitting out of bed. CSF culture grew gram-negative cocci, which was identified as *Neisseria meningitidis *group B, type NT, subtype NT P1.16/nt. She underwent contact tracing and completed a 10-day course of intravenous ceftriaxone. She continued to make a slow but progressive recovery. After a period of rehabilitation and intense physiotherapy she was discharged home 40 days after admission, with mild residual gait ataxia.

## Conclusions

This case report presents two important clinical concepts: firstly, the presentation of chronic meningitis and secondly, the clinical presentation of bacterial meningitis in the older adult (defined as > 60 years old). The diagnosis was delayed due to the highly atypical clinical presentation [1].

Chronic meningitis is defined as symptoms and signs of meningeal inflammation and persisting cerebrospinal fluid (CSF) abnormalities such as elevated protein level and pleocytosis for at least one month [2,3]. It affects less than 10% of meningitis sufferers and is linked to a large variety of both infective and non-infective causes [4]. However, whilst there are numerous published individual case reports on chronic meningitis, there is a definite paucity of large case series in the literature. The most common cause of chronic meningitis is Mycobacterium tuberculosis, which accounts for 40–60% of cases [3,5]. Other relatively frequent causes include malignancy (8–13%) and crytococcal infection 7–11%) [3,5]. In up to 33% of cases no underlying cause is identified [3,5]. Chronic meningococcal meningitis is rare and is limited to a few isolated case reports in the literature [6-8].

There are several distinguishing features that may help to differentiate chronic meningitis from adult acute bacterial meningitis (table 1). The classic triad of clinical features of meningitis (fever, neck stiffness, altered mental state), whilst seen in up to 85% of patients presenting with acute bacterial meningitis is far less commonly seen in chronic meningitis [3,5,9]. Focal neurological signs with cranial nerve palsies and abnormal CT brain findings are also far more commonly seen in chronic meningitis [5,10].

**Table 1:** Features distinguishing chronic meningitis (bacterial and non bacterial) compared with acute bacterial meningitis

**Description**	**Acute bacterial meningitis**	**Chronic meningitis**
**Aetiology**	Variable Neisseria meningitides 13–56% [10,17, 39] Streptococcus pneumoniae 24–37% [10,17]	Variable TB- 40–60% [3,5] Malignancy 8–13% [3,5] Cryptococcus 7–11% [3,5] Unknown 30–33% [3,5]
**Clinical features**		
**- Classic triad of fever, headache and neck stiffness**	85% [9]	10% [4]
**- Fever**	78–91% [39]	44% [4]
**- Headache**	32–68% [39]	79% [4]
**- Neck Stiffness**	58–82% [39]	75% [5]
**- Altered Mental state**	52–82% [39]	41% [4]
**- Focal neurology**	23% [39]	32% [5]
**- Papilloedema**	<1–4% [9,10]	30% [5]
**- Cranial Nerve Palsies**	4% [10]	24% [5]
**Mortality**	Variable – aetiology dependent 19.7–25% overall [10,17] 37–44% ≥ 60 years old [1,10] 10–25% < 60 years old [10,16–20]	Variable – aetiology dependent 29%- overall [5]
**Elevated WCC, CRP and ESR**	Elevated	Normal or only mildly elevated [5]
**Hyponatraemia**	<10%	>90% [5]
**Cerebrospinal fluid analysis**	10% – lymphocytic [9,17] 90% – neutrophilic [9,17] Gram stain positive 57–90% [9,10,17]	>90% lymphocytic [5] <10% neutrophilic [5] Gram stain positive <10% [5]
**Abnormal CT**	2.7 – 13% [10,40]	60% [5]

WCC, white cell count; CRP, C-reactive protein; ESR, erythrocyte sedimentation rate.

Hyponatraemia (as in our patient), whilst very uncommon in acute bacterial meningitis, is seen in the vast majority of cases of chronic meningitis [5,11]. Although there was a persistent mild neutrophilia, both the CRP and ESR were normal throughout the course of the disease, which, whilst being highly unusual for acute meningitis, has been reported, in chronic meningitis [12,13].

Acute bacterial meningitis is usually a rapidly progressive and highly lethal disease in older adults [1]. Rapid diagnosis is vital as the prognosis worsens with treatment delay leading to a high rate of sustained neurological deficit in this age group [14,15]. Despite the widespread use of antibiotics the overall case mortality rate remains unchanged and is far higher (37–44%) in the older adult compared with that seen in younger adults (10–25%) with significant long-term morbidity (up to 70% of infected patients) in survivors [16-20].

Given the success of childhood immunization, and an increasingly aging population, the proportion of older adults presenting with bacterial meningitis is increasing [16]. There are several additional factors, which make the older adult more prone to bacterial meningitis. Older adults often have underlying acute and chronic diseases (e.g. diabetes, renal or hepatic failure) with immunosenescence (age related decline in immune function) [1,21]. This can lead to symptoms, which can be confused with those of meningitis and at the same time increase the propensity to infection [1,21]. The role of immunosenescence in predisposing patients to bacterial meningitis is not clearly defined, but appears to relate to defects in innate, specific cellular and humoral immunity leading to an attenuated immune response [1,22-24]. Persons who lack or are deficient of antibody-dependent, complement-mediated lysis (bacteriocidal activity) are most susceptible to meningococcal disease [25]. Our patient had an unremarkable past medical history with normal complement and immunoglobulin levels with no evidence of immunosuppresion [26-28].

The clinical presentation of bacterial meningitis is more variable in the older as compared with the younger adult, with fewer patients manifesting with the classic symptoms of fever, neck stiffness and altered mental state than among younger adults [1]. It has been suggested that 1 of 3 findings (fever, neck stiffness, altered mental state) is present in virtually all patients with meningitis and that the absence of these features virtually excludes meningitis with a high negative predictive value (table 1) [1]. Our patient had none of these features on presentation and had been unwell for four weeks prior to presentation, but did develop a mild fever (<38°C) and cognitive dysfunction during her inpatient stay. The blunted febrile response is well recognised in older adults in general [29].

Our patient's CSF showed a lymphocytosis, raised protein, and low glucose ratio, which are seen in only 10% of bacterial meningitis cases. This CSF profile would normally suggest infection with *Listeria monocytogenes *meningitis or alternative causes such as tuberculous and fungal infection [30,31].

*Neisseria (N) meningitidis *is a leading cause of bacterial meningitis in the Western World and tends to predominate in young adults [19,20,25]. *N. meningitidis *is a gram-negative, aerobic diplococcus. It is classified into serogroups (e.g. A,B,C etc) according to the immunological reactivity of their polysaccharides [25]. The most prevalent serogroups implicated in clinical meningococcal meningitis are serogroup B (62%, as in our patient) and the more virulent serogroup C (22%) [19,20]. The relatively reduced virulence of serogroup B may partly explain the chronicity of presentation and reduced inflammatory response seen in our patient. Serogroups B and C have a seasonal variation occurring more commonly in the first quarter of the year (our patient presented in February) [19]. Meningococcal meningitis is also more common among the following groups: persons of black race; lower socioeconomic classes; those exposed actively or passively to tobacco smoke; persons exposed to overcrowding and amongst binge drinkers [32-37].

This case highlights the diagnostic challenge associated with bacterial meningitis presenting in an older patient. The presentation was made even more difficult owing to the blunted febrile response, the lack of inflammatory response observed in laboratory tests and the chronicity of the patient's symptoms. The diagnosis required thorough investigation during the inpatient stay. Early lumbar puncture is to be encouraged as it is essential to confirm the diagnosis. Despite a delayed diagnosis appropriate antibiotic therapy can still lead to a good outcome.

## Competing interests

The authors declare that they have no competing interests.

## Authors' contributions

MD generated the idea of writing the case report and was the consultant in charge of the patient. CD reviewed the case notes of the patient and wrote the original draft of the case presentation. CB significantly revised the original draft and added the conclusions, references and figures. AH offered considerable help with the manuscript revisions. All authors contributed to the final version of the manuscript.

## Pre-publication history

The pre-publication history for this paper can be accessed here:


